# Effect of Restraint Stress on Pain Sensitivity, Spinal Trigeminal Nucleus Neurons, and Astrocytes in the Masseter Area of Rats

**DOI:** 10.1155/2022/2345039

**Published:** 2022-08-05

**Authors:** Feng Han

**Affiliations:** Department of Human Anatomy, Jilin Medical University, Jilin City 132013, Jilin Province, China

## Abstract

To explore the changes of pain sensitivity (PS) in the masseter area (MA) in the rat model of psychological stress and the mechanism of action between spinal nucleus neurons and astrocytes in the trigeminal ganglion. The 40 Sprague-Dawley rats were randomly divided into control group (no treatment), group A (restraint stress (RS) 1 d), group B (RS 7 d), and group C (RS 14 d). The body weight growth rates (WGR) of rats in each group were compared and the difference of CORT and ACTH in serum was analyzed by ELISA. The open field test and the elevated “cross” maze test were adopted to detect the behavioral changes of rats. Finally, pain threshold of the MA in rats, the activation amount of brain tissue medulla oblongata parts astrocytes markers Glial fibrillary acidic protein (GFAP), and the protein expression of IL-1*β* and IL-1RI were detected. The results showed the WGR at 7 d and 14 d was greatly lower than control group (*P* < 0.01). In addition, the activity level and serum CORT and ACTH levels AND mean pain threshold in the MA of groups B and C were greatly lower than control group (*P* < 0.05). The activation rate of GFRP in group C (*P* < 0.01) and the protein expression of IL-1*β* and IL-1RI (*P* < 0.05) in rat trigeminal ganglion astrocytes of groups B and C was greatly higher than control group, indicating the increase of RS time, the release of IL-1*β* and IL-1RI can activate neurons and astrocytes in spinal trigeminal nucleus (STN) nerve and increase the PS of the MA.

## 1. Introduction

With the development of society and the acceleration of the pace of life, the role of psychological stress in various diseases has become more and more prominent. Psychological stress has been reported to be a common risk factor for 75% to 90% of diseases [1].

Stress can stimulate a series of information transmission in the peripheral nervous system and the central nervous system. It is the process of the body dealing with external stimuli and challenges, resulting in a nonspecific adaptive mechanism, including environmental and psychological factors. Acute psychological stress can induce the body's natural immune system, enhance resistance to pathogenic microbial infection, and speed up wound healing. Chronic psychological stress will break the homeostasis of the internal environment, change the function of immune cells, lead to immune dysfunction, and induce a variety of physical and mental diseases, such as depression [2].

At present, there are many methods used to build stress animal models, mainly by directly exposing animals to the stress environment that cannot be avoided to make them produce corresponding stress response. When the body is stressed at the psychological level, it will activate the hypothalamic–pituitary–adrenal axis, thereby promoting the secretion of adrenocorticotropin by the pituitary gland and increasing the secretion of glucocorticoid in the body [3]. However, studies have shown that the occurrence and development of a large number of diseases are related to psychological stress, such as mental diseases like epilepsy, peptic ulcer, hypertension, rheumatoid diseases. [4]. Therefore, the establishment of stress animal models is of great significance for us to study the occurrence and development of related diseases. The methods of building animal models of psychological stress include the following: restraint method, forced swimming method, punitive drinking water method, etc. Among them, restraint method is a method that does not cause physical damage to animal models [5]. The stress animal model after construction can be verified by open field test, elevated “cross” maze test, and sugar and water consumption tests.

Pain is a series of complex stress responses produced by the organism after the organism is stimulated by injury and often causes the individual to be depressed, slow in response, sympathetic excitement, and other reactions. Peripheral sensitization and central sensitization play an important role in the occurrence and development of pain [4]. According to statistics, the pain of the mouth and maxillofacial is one of the common and high-incidence parts of human pain diseases, among which the trigeminal nerve distributed in the maxillofacial tissue in the form of free nerve endings can feel the stimulation of injury. When the trigeminal nerve primary afferent nerve fiber transmits the pain to the sensory nucleus composed of the trigeminal nerve sensory main nucleus and spinal trigeminal nucleus (STN) in the brain stem, it conducts the pain conduction, and the STN neuron nerve plays a key role in the central sensitized process of the pain transmission system [6]. The nervous system is made up of neurons and glial cells, and astrocytes located in central nervous cells are also closely related to pain. A large number of studies have shown that GFAP, an astrocyte marker, is upregulated in the established masseter palpation model [7].

Currently, studies have shown that the body is stimulated by continuous low-intensity psychological stress, which will lead to the appearance of pain sensitivity (PS) in the mouth and maxillofacial region [8]. However, by constructing the rat model of psychological stress, the study on the effect of psychological stress on the PS of the masseter area (MA), STN neurons, and astrocytes in rats is less. Therefore, a rat psychological stress model was constructed with the restraint method, and the model was verified based on the open-field test, and the “cross” maze test was elevated, and then the influence of the increased stress time was tested on the pain threshold of the masseter area and the expression of glial fibrillary acidic protein (GFAP) in the brain tissue. The purpose of this study is to lay a foundation for the study of the mechanism of action of psychological stress on STN neurons and astrocytes during the nociceptive sensitivity of the MA in rats.

## 2. Materials and Methods

### 2.1. Establishment of Experimental Animal and RS Model

In this research, 40 SD male rats purchased from Shanghai Slack Laboratory Animal Co., LTD was selected as subjects. The average weight of all the rats was about 215 ± 10 g. The rats were kept in separate cages in the animal feeding laboratory, and the average temperature and relative humidity were kept at 23 ± 2 and 60 ± 5%, respectively. All rats were fed standard feed, given natural light for 12 hours a day, and all rats were free to eat.

The freedom of movement of SD rats in the experimental group was restricted by using a cylindrical restraint device with a height of about 30 cm and a diameter of 10 cm made of elastic metal mesh, and the diameter of the restraint device was adjusted according to the growth condition of the experimental rats to ensure that the body of the rats was not subjected to physical pressure. Forty SD rats were randomly divided into four groups: control group, test group A (RS 1 d), test group B (RS 7 d), and test group C (RS 14 d), with 10 rats in each group. The rats in the control group were kept in cages with no treatment every day, from 9:00 a.m. to 5:00 p.m.

### 2.2. Evaluation of Captive Stress Animal Models

Weight detection: rats in the four groups were weighed at 1, 7, and 14 days after RS stimulation and the WGR of the control group and the experimental group were calculated.

Detection of serum stress hormone content: on day 1, day 7, and day 14, rats were anesthetized by intraperitoneal injection of 1% pentobarbital sodium 50 mg/kg. A quantity of 1.5 ml of blood was taken from the heart and placed on ice for 30 min, then centrifuged at 3000 rpm for 15 min, and the supernatant was taken. Avidin-biocomplex-Enzyme-Linked Immunosorbent Assay (ABC-ELISA) was used to determine the content of CORT and ACTH in serum of rats. Monoclonal antibodies of rat CORT and ACTH were coated in a 96-well plate and were mixed with 100 *μ*L/well standard substance or serum to be tested, and then placed at 37°C for 2 h. After washing the reaction plate for five times, filter paper was used to print the dry reaction plate. The mixture was mixed with 100 *μ*L/hole and placed at 37°C for 1 h. After washing the reaction plate, 100 *μ*L/hole was added into the enzyme-labeled antibody and placed at 37°C for 30 min. After washing reaction plate, 100 *μ*L/hole working fluid was added to mix well, placed at 37°C to avoid light for 30 minutes. The final solution of 100 *μ*L/hole was mixed, and the absorbance value at 450 nm was detected in the microplate reader.

Open field test: rats were placed in a 100 × 100 × 80 cm open field test box in a low-light and sound-proof room on 1 d, 7 d, and 14 d after RS stimulation. After the rats were acclimated for 3 min, the total range (cm), the central range (cm), and the velocity (cm/s) of the rats were recorded within 15 min. After the experiment was completed, the chamber needed to be cleaned and 75% ethanol sprayed to treat the residual odor in the chamber.

Elevated “cross” maze test: rats were placed in the center of the “cross” structure made of opaque material about 70 cm above the ground at 1 d, 7 d, and 14 d after RS stimulation (upper and lower ends were open arms and left and right segments were closed arms. The wall length was 50 cm and the width was 10 cm), and the activity of the rats was recorded by camera within 5 min after the insertion. The final results were expressed as the percentage of retention time (%) and the percentage of retention times (%) in the open arm of each rat. After the experiment was completed, the chamber needed to be cleaned and 75% ethanol sprayed to treat the residual odor in the chamber.

### 2.3. Detection of PS of MA in Rats

The rats were placed in a quiet and closed space for 20 minutes prior to the restraint of stress stimulation, and for 1, 7, and 14 days after stimulation. Then, an electronic pain-measuring device was used to stimulate the MA about 1 cm below the middle line between the eyes and ears of the rats. When the rats showed three or more times of avoidance, cowering, and vocalization, it indicated that the rats had pain response. At this time, the pressure value of the electronic pain-measuring device was recorded, and the mean value of five times of stimulation was taken as the pain threshold of the rat.

### 2.4. Glial Fibrillary Acidic Protein Detection in the Rat Trigeminal Ganglion

The rats were anesthetized with 1% pentobarbital sodium (50 mg/kg) intraperitoneally at 1 d, 7 d, and 14 d after RS stimulation. The rats were placed in a supine position. Perfusion needle was used to rise from the left ventricle of the rat to the aorta, and the prepared mixture of 4% paraformaldehyde, 0.2% saturated picric acid, and 0.1 mol/L PB was added. About 3 h later, the rat brain tissue was taken out for fixation and dehydration and placed in sucrose solution. After the brain tissue had sunk to the bottom, the medulla oblongata in the brain tissue was cut into sections 30 *μ*m thick and placed in 0.01 mol/L phosphate buffer (PBS). Sections were removed and washed with 0.01 mol/L PBS for 3 times × 10 min. Donkey serum was added and incubated at room temperature for 30 min. The 1 : 5000 diluted monoclonal mouse Glial fibrillary acidic protein (GFAP) a fight and the 1 : 5000 diluted monoclonal rabbit anti-neun primary antibody were added, respectively, and incubated in warm shaker overnight. Washed with 0.01 mol/L PBS for 3 times × 10 min, 1 : 500 diluted donkey anti-mouse IgG secondary antibody was added and incubated at room temperature for 3 h. After being washed with 0.01 mol/L PBS for 3 times × 10 min, the staining results were observed under a laser confocal microscope after sealing, and Imag-Pro Plus image analysis software was used to process the results.

### 2.5. Analysis of Protein Expression of IL-1*β* in Trigeminal Ganglion Astrocytes

The rat trident ganglion tissues obtained earlier were placed in the EP tube containing phenylmethylsulfonyl phosphating solution and placed in the icebox for enough tissue lysis. It was placed in a centrifuge and centrifuged at 12000 rpm for 3 min, then supernatant was taken. BCA kit (Shanghai Biyuntian Biotechnology Co., LTD., China) was used to determine protein concentration; the configured gel was fixed in the electrophoresis tank, and 6 *μ*L samples were added to each well to be tested; finally, PVDF membrane was used for transfer printing. After that, TBST solution was used for rinsing and sealing for 2 h. After rinse, 1 : 500 dilution of rabbit anti-IL-1*β* I, 1 : 100 dilution rabbit anti-IL-1RI, and 1 : 5000 dilution rabbit anti-*β*-actin I were added, respectively, incubated at 4°C overnight. After rinsing, a 1 : 500 diluted donkey antirabbit secondary antibody was added and incubated at room temperature for 1 h. After rinsed, they were developed in a darkroom, and protein expression was detected by BeyoECL.

### 2.6. Statistics Process

SPSS19.0 software was used to conduct statistical processing of all data, and the data were misrepresented by mean ± standard, and Duncan multiple comparisons were used in ANOVA analysis for intergroup comparisons. When *P* < 0.05, the difference between the two groups was considered statistically significant.

## 3. Results

### 3.1. Effect of RS on Body Weight in Rats

**Figure 1:**
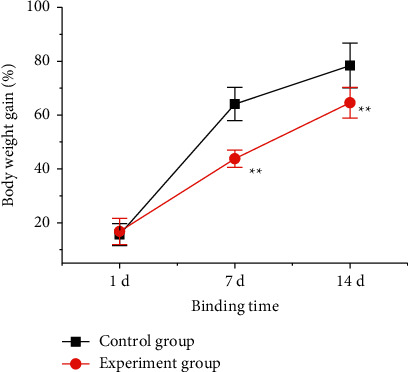
The WGR difference of rats after different time-RS stimulation. Note: ^*∗∗*^ meant there was an extremely SSD compared with the control group, *P* < 0.01.

In order to explore the influence of RS at different times on the weight of rats, the changes in the WGR of rats in the control group and the experimental group at 1, 7, and 14 days after RS stimulation were compared, and the results were shown in Figure 1. It showed that there was no statistically significant difference (SSD) in the WGR of rats when the stress stimulation was restrained for 1 d (*P* > 0.05). On the 7 d RS stimulation, the WGR of the control group was 64.1 ± 6.2%, while that of the experimental group was 43.8 ± 3.2%, and the WGR of the experimental group was greatly lower than that of the control group (*P* < 0.01). However, when the stress was restrained for 14 d, the WGR of the control group was 78.4 ± 8.3%, while that of the experimental group was 64.6 ± 5.7%, and the WGR of the experimental group was greatly higher than that of the control group (*P* < 0.01).

### 3.2. Effects of Bondage Stress on CORT and ACHT in the Serum of Rats

**Figure 2:**
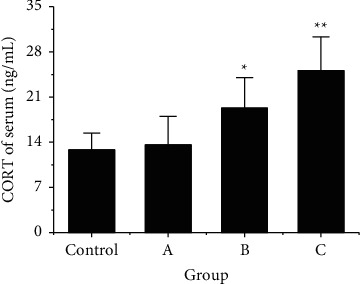
Comparison of CORT content in the serum of rats after RS. Note: ^*∗*^ meant there was a SSD compared with the control group, *P* < 0.05; ^*∗∗*^ indicated that there was an extremely SSD compared with the control group, *P* < 0.01.

In order to explore the influence of RS at different times on serum-related indexes of rats, the differences in CORT and ACHT contents in the serum of the four groups of rats were detected and compared, and the results were shown in Figure 2. According to Figure 2, CORT content in blood of rats in the control group was 12.8 ± 2.6 ng/mL, rats in group A were 12.6 ± 4.4 ng/mL, rats in group B were 19.3 ± 4.7 ng/mL, and rats in group C were 25.1 ± 5.2 ng/mL. CORT content in serum of group B was greatly higher than that of control group (*P* < 0.05). The content of CORT in serum of group C was greatly higher than that of control group (*P* < 0.01).

**Figure 3:**
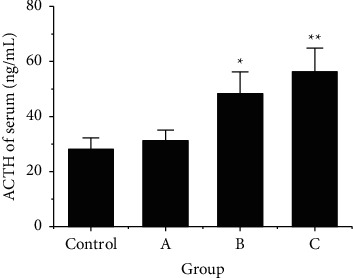
Comparison of ACTH content in the serum of rats after RS. Note: ^*∗*^ meant there was a SSD after comparison with the control group, *P* < 0.05; ^*∗∗*^ indicated that there was an extremely SSD after comparison with the control group, *P* < 0.01.

According to Figure 3, the ACHT content in blood of rats in the control group was 28.1 ± 4.2 ng/mL, rats in group A were 31.2 ± 3.9 ng/mL, rats in group B were 48.3 ± 7.9 ng/mL, and rats in group C were 56.2 ± 8.7 ng/mL. The content of ACHT in serum of group B and group C was greatly higher than that of control group (*P* < 0.01).

### 3.3. Effects of Bondage Stress on Behavior in Rats

In order to compare the effects of bondage stress on the behavior of rats, open field test and elevated “cross” maze test were used to detect the behavioral changes of rats under different bondage stress. According to Table 1, in the open field test, there was no SSD between the total activity distance, central activity distance, and activity speed between the control group and group A (*P* > 0.05). The total active distance, central active distance, and active speed of rats in group B were greatly lower than those in control group (*P* < 0.05). The total active distance, central active distance, and active speed of group C were greatly lower than those of control group (*P* < 0.01). In the elevated “cross” maze test, there was no SSD between the control group and group A in the percentage of open-arm retention time and the percentage of open-arm retention times (*P* > 0.05). However, the percentage of open-arm retention time and the percentage of open-arm retention time in group B were greatly lower than those in the control group (*P* < 0.05). The percentage of open-arm retention time and open-arm retention times in group C was greatly lower than that in control group (*P* < 0.01).

### 3.4. Effect of RS on the PS of MA in Rats

**Figure 4:**
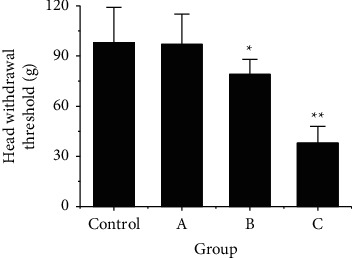
Comparison of the mean threshold of PS in the MA in rats after RS. Note: ^*∗*^meant there was a SSD after comparison with the control group, *P* < 0.05; ^*∗∗*^indicated that there was an extremely SSD after comparison with the control group, *P* < 0.01.

In order to compare the effects of different RS on the PS of the MA in rats, an electronic pain-measuring device was used to detect the mean pain threshold of the MA in the control group and rats with RS stimulation on 1 d, 7 d, and 14 d, and the results were shown in Figure 4. According to Figure 4, the mean threshold of pain in the MA of rats in the control group was 98 ± 21 g, the mean threshold of pain in the MA of rats in group A was 97 ± 18 g, the mean threshold of pain in the MA of rats in group B was 79 ± 9 g, and the mean threshold of pain in the MA of rats in group C was 38 ± 10 g. After comparison, it showed that there was no SSD between the mean threshold of pain in group A and control group (*P* > 0.05), the mean threshold of pain in group B was greatly lower than that in control group (*P* < 0.05), and the mean threshold of pain in group C was greatly lower than that in control group (*P* < 0.01).

### 3.5. Immunofluorescence Staining of GFAP in STN Nerve in Rats after RS

**Figure 5:**
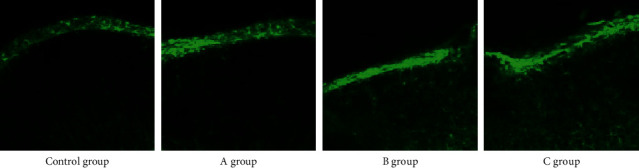
GFAP immunofluorescence staining in STN nerve in rats after RS.

In order to compare the effects of different time-RS on GFAP in STN in rats, immunofluorescence staining technology was used to detect the staining of GFAP in STN in brain tissues of rats. As shown in Figure 5, only a small number of GFAP positive neurons were found in the control group. The positive expression of GFAP in STN of rats in group A increased, while that in group B and group C increased greatly.

**Figure 6:**
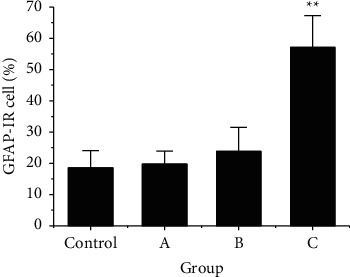
The activation rate of GFRP in STN of rats after RS. Note: ^*∗∗*^indicated a very SSD after comparison with the control group (*P* < 0.01).

The activation rate of GFAP in STN nerve in rats was compared. As shown in Figure 6, the activation rate of GFAP in STN of rats in group C was greatly higher than that in the control group (*P* < 0.01); however, there was no SSD in the activation rate of GFAP in STN in the control group, group A, and group B (*P* > 0.05).

### 3.6. The Expression of IL-1*β* Protein in Astrocytes Cells of Trigeminal Ganglion after RS

**Figure 7:**
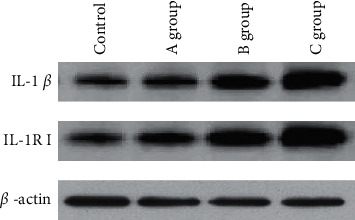
Western blot results of genes *IL-1β* and *IL-1RI* in rat trigeminal ganglion astrocytes.

The protein expression changes differences of IL-1*β*, IL-1R I, and reference genes *β-actin* in rat trigeminal ganglion astrocytes of each group were compared. From Figure 7, it can be found intuitively that the protein expression level of *β*-actin in star glial cells of trigeminal ganglia of rats in each group was nearly the same, while the protein expression of genes *IL-1β* and *IL-1RI* was the lowest in the control group and highest in group C.

**Figure 8:**
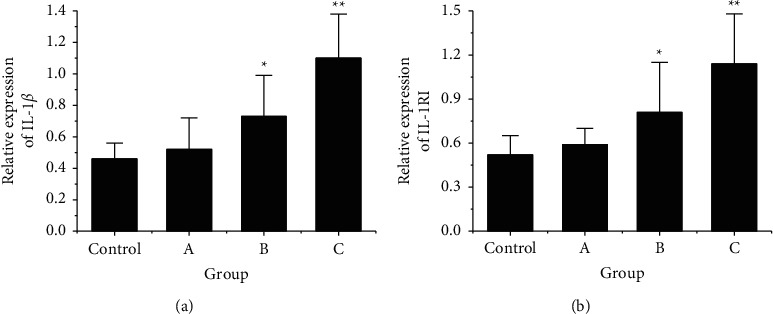
The quantitative results of IL-1*β* and IL-1RI proteins in rat trigeminal ganglion astrocytes. Note: (a) was the quantitative result of IL-1*β* protein expression; (b) was the quantitative result of IL-1RI protein expression; ^*∗*^ indicated that there was SSD after comparison to the control group, *P* < 0.05; ^*∗∗*^ indicated that there were extremely SSDs after comparison to the control group, *P* < 0.01.

The protein expression differences of genes *IL-1β* and *IL-1RI* in rat trigeminal ganglion astrocytes of each group were quantitatively analyzed. As shown in Figure 8, with the increase of the RS time, The protein expression of IL-1*β* in star glia cells of trigeminal ganglia showed a gradually increasing trend in each group, and the expression of IL-1*β* in group B was greatly higher than that in the control group (*P* < 0.05), and that in group C was greatly higher than that in the control group (*P* < 0.01). As shown in Figure 8(b), with the increase of the RS time, the expression of IL-1RI protein in star glial cells of trigeminal ganglia of rats in each group also showed a trend of gradual increase, and the expression of il-1ri protein in rats in group B was greatly higher than that in the control group (*P* < 0.05), and the expression of IL-1RI protein in rats in group C was greatly higher than that in the control group (*P* < 0.01). However, there was no SSD in IL-1*β* and IL-1RI protein expressions between the control group and group A (*P* > 0.05).

## 4. Discussion

In recent years, the methods used to construct animal models of psychological stress include electric foot stimulation, thermal stimulation, compulsive swimming treatment, restraint method, and so on, the stress model obtained by the restraint method can cause a typical nonspecific stress response in animals and does not cause direct trauma to the animal body [9, 10]. In this research, stress animal models were obtained by a restraint method. Results were found with the increase of bound time, and the model rats showed slow downward movement and WGR, and bondage stress model of rats serum levels of stress hormones. CORT and ACTH also increased with the increase of stress time reduce, indicating that the rat model with the constraints of this study to construct stress has been in a state of stress [11]. Studies have shown that psychological stress can affect the emotion-related hippocampus and other tissues in the brain of rats, thereby causing behavioral changes in rats [12]. In this research, under the open-field test and the elevated “cross” maze test, it showed that with the increase of the stress time, the activity level of the rat model with RS decreased greatly, indicating that the rats had negative anxiety emotions, which was consistent with the results of Xin et al. [13].

The activity of atpase in the masseter of rats decreases gradually after they are subjected to psychological stress, which can increase the sensitivity of masseter myodynamia [14]. In this research, it showed that the mean pain threshold in the MA of rats decreased with the increase of stress time after stress stimulation was restricted, indicating that psychological stress would lead to the increase of PS in the MA of rats, which was consistent with Muzalev's research results [15]. The reason for this phenomenon may be that when the rats are in a state of stress, they would increase the secretion of hormones such as epinephrine in their bodies, which would lead to local hypoxia and ischemia after increased muscle activity. The caudal subnucleus of the STN played an important role in the transmission of pain in the oral and maxillofacial regions. Okamoto et al. found that when the rats were subjected to compulsive swimming stress, the pain threshold of the mouth and maxillofacial region decreased, and the activation degree of neurons in the caudal subnucleus of the STN was significant increased [16]. When the astrocytes in the STN were activated, the specific markers of GFAP expression would obviously rise [17]. This is consistent with the findings in this research that the proportion of GFAP positive neurons in the brain tissues of rats increased greatly with the increasing time of RS, indicating that astrocytes and neurons in the trigeminal nerve were greatly activated, which indicates that with the passage of stress time, the activation of astrocytes and neurons in the trigeminal nerve will become more and more obvious [18]. The relationship between astrocytes and neurons in trigeminal ganglion is an important part of nerve signal regulation. The activated glial cells release a series of inflammatory factors, such as interleukin-1 (IL-1*β*), which bind to the relevant receptors on the surface of the neuron, thereby enhancing the physiological activity of the neuron and finally aggravating the sensitivity of maxillofacial pain [19, 20]. It was found that with the increase of stress time, the protein expression of IL-1*β* and IL-1RI in rat trigeminal ganglion astrocytes increased greatly at 14 d of the stress, indicating that the release of IL-1*β* and IL-1RI can activate astrocytes, which in turn increases the sensitivity of the masseter pain in rats, which was consistent with the research results of Doyle et al. [21].

In this research, the RS method was used to construct rat models with different stress times. The stress rat model was verified by open-field test and elevated “cross” maze test. Subsequently, the changes in PS in the MA and the expression of astrocyte-specific marker GFAP in the brain tissues of the rat model were detected under different RS times. The results showed that the rat model of psychological stress was successfully constructed by restraint method, and the PS of the MA in the rat model increased with the increase of stress time. Finally, it was found that the STN neurons and astrocytes in rat brain may be activated due to the increase of the release of IL-*β* and IL-1RI. However, only basic research was constructed, and the molecular regulatory mechanism that increases the sensitivity of masseter myalgia in rats after stress stimulation needs to be further studied. In conclusion, the results can lay a foundation for the subsequent research on the PS of MA caused by psychological stress.

## Figures and Tables

**Table 1:** Comparison of behavioral changes in rats after RS (mean ± standard).

Group	Open field test			Elevated “cross” maze test	
	Total trip (cm)	Central route (cm)	Activity speed (cm/s)	Percentage of open-arm retention time (%)	Percentage of open-arm retention (%)

Control group	4011 ± 102	126 ± 20	4.8 ± 0.61	40 ± 11	39 ± 7
Group A	3824 ± 110	113 ± 18	4.7 ± 0.43	36 ± 10	33 ± 10
Group B	2936 ± 127^*∗*^	76 ± 19^*∗*^	3.6 ± 0.71^*∗*^	24 ± 7^*∗*^	26 ± 8^*∗*^
Group C	2538 ± 109^*∗∗*^	61 ± 12^*∗∗*^	2.8 ± 0.52^*∗∗*^	21 ± 9^*∗∗*^	23 ± 9^*∗∗*^

Note: ^*∗*^meant there was a SSD after comparison with the control group, *P* < 0.05; ^*∗∗*^indicated that there was an extremely SSD after comparison with the control group, *P* < 0.01.

## Data Availability

The datasets used and analyzed during the current study are available from the author upon reasonable request.
